# p73α1, an Isoform of the p73 Tumor Suppressor, Modulates Lipid Metabolism and Cancer Cell Growth via Stearoyl-CoA Desaturase-1

**DOI:** 10.3390/cells11162516

**Published:** 2022-08-13

**Authors:** Zachary Rabow, Kyra Laubach, Xiangmudong Kong, Tong Shen, Shakur Mohibi, Jin Zhang, Oliver Fiehn, Xinbin Chen

**Affiliations:** 1 West Coast Metabolomics Center, University of California, Davis, CA 95616, USA; 2 Comparative Oncology Laboratory, Schools of Medicine and Veterinary Medicine, University of California, Davis, CA 95616, USA

**Keywords:** p73 isoforms, the p53 family, Stearoyl-CoA Desaturase, lipid metabolism, Kennedy pathway

## Abstract

Altered lipid metabolism is a hallmark of cancer. p73, a p53 family member, regulates cellular processes and is expressed as multiple isoforms. However, the role of p73 in regulating lipid metabolism is not well-characterized. Previously, we found that loss of p73 exon 12 (*E12*) leads to an isoform switch from p73α to p73α1, the latter of which has strong tumor suppressive activity. In this study, comprehensive untargeted metabolomics was performed to determine whether p73α1 alters lipid metabolism in non-small cell lung carcinoma cells. RNA-seq and molecular biology approaches were combined to identify lipid metabolism genes altered upon loss of *E12* and identify a direct target of p73α1. We found that loss of *E12* leads to decreased levels of phosphatidylcholines, and this was due to decreased expression of genes involved in phosphatidylcholine synthesis. Additionally, we found that E12-knockout cells had increased levels of phosphatidylcholines containing saturated fatty acids (FAs) and decreased levels of phosphatidylcholines containing monounsaturated fatty acids (MUFAs). We then found that p73α1 inhibits cancer cell viability through direct transcriptional suppression of Stearoyl-CoA Desaturase-1 (SCD1), which converts saturated FAs to MUFAs. Finally, we showed that p73α1-mediated suppression of SCD1 leads to increased ratios of saturated FAs to MUFAs.

## 1. Introduction

Lipids are key building blocks in cells that are essential for membrane formation, energy storage, and cell signaling. In particular, glycerophospholipids are the primary component of cell membranes and are composed of a phosphate head group attached to a diacylglycerol (DG) backbone and two fatty acids (FAs) [1]. There are multiple classes of glycerophospholipids, but phosphatidylcholines (PCs) are the most abundant class within eukaryotic cell membranes, contributing up to 50% of the total phospholipid content [2]. PC biosynthesis occurs through the Kennedy pathway (consisting of the CDP-choline and CDP-ethanolamine branches), the Lands Cycle, or the phosphatidylethanolamine methyltransferase (PEMT) pathway [3,4,5]. The Kennedy pathway is initiated by intracellular choline import via choline transporters (CTLs and CHTs), where choline is quickly converted to phosphocholine (P-choline) by choline kinases in the cytosol [6]. P-choline is then converted to CDP-choline by CDP-choline synthetase in the nucleus or cytosol. Finally, the choline head group is linked to a DG backbone by choline/ethanolamine phosphotransferase to form PCs [3]. In the Lands Cycle, phospholipases cleave PCs to form lysophosphatidylcholines (LPCs) and FAs [7]. LPCs can then be converted back to PCs through lysophospholipid acyltransferases. The PEMT pathway, occurring in the liver, methylates phosphatidylethanolamines (PEs), through the methyl donor S-adenosylmethionine, to form PCs [8]. Various PC metabolites exist due to differences in fatty acyl chain length and saturation [9].

Traditionally thought of as membrane lipids, the role of PCs in energy metabolism, cell signaling, and lipoprotein transport is becoming more apparent [10].

Altered lipid metabolism has become a consequential hallmark of tumorigenesis [11]. Carcinogenic cells can increase lipid synthesis and/or uptake to sustain their rapidly dividing state [12]. Evidence shows that increased levels of PCs and other choline-containing metabolites are associated with oncogenesis [13]. Cancer cells maintain high levels of PC synthesis through increased choline import and the formation of PC intermediates [14,15,16]. An abundance of PCs and choline-containing metabolites confers cancer cell survival in metabolically demanding conditions [17]. PCs can also be modified to form signaling lipids, such as arachidonic acid, phosphatidic acid, and DGs. These signaling lipids activate a myriad of oncogenic pathways to promote tumorigenesis [18,19]. For example, phosphatidic acid activates the mTOR pathway to inhibit apoptosis and promote cancer cell survival [20]. Similarly, arachidonic acid and its derivatives have been shown to promote angiogenesis, and tumor cell proliferation and invasion [21]. As such, altered PC metabolism is undoubtedly critical for driving tumorigenesis.

p73, a member of the p53 family of tumor suppressors, is a transcription factor and regulates many cellular processes [22,23]. The p73 gene structure affords the formation of multiple isoforms with varying functions. The N-terminal isoforms arise from two promoters, denoted P1 and P2, located upstream of exon 1 and in intron 3, respectively [24]. Transcription initiation from the P1 promoter leads to the expression of TAp73 isoforms [24], which have a tumor suppressive function similar to that of p53 [25,26,27,28]. Conversely, the P2 promoter produces ΔNp73 isoforms [29] that promote cell survival and can function as oncoproteins [30,31,32]. At the 3′ end, alternative splicing of exons 11, 12 and 13 gives rise to several known C-terminal isoforms [29,33,34]. p73α is the major isoform expressed in most human and mouse tissues and the most well-studied [35,36]. Previously, we found that exclusion of exon 12 (*E12*) leads to an isoform switch from p73α to a novel isoform that we termed p73α1 [37]. We found that p73α1 was endogenously expressed in multiple cancer cell lines, as well as normal and cancerous human prostate tissues [37]. Additionally, we found that p73α1 inhibits cancer cell viability in vitro, and mice deficient in *E12* are not prone to spontaneous tumors [37]. To further investigate the biological function of p73α1, we wanted to explore whether p73α1 regulates lipid metabolism.

In the present study, we investigated the effect of p73α1 expression on the lipidome in non-small cell lung carcinoma (H1299) cells, which do not express p53. We found that loss of *E12* leads to decreased levels of PCs, PEs, and their derivatives. RNA-seq analysis showed that E12-knockout (E12-KO) cells had decreased expression of several enzymes involved in PC and PE synthesis, but choline import appeared unchanged. Furthermore, we found that loss of *E12* led to increased levels of PCs containing saturated FAs and decreased levels of PCs containing mono-unsaturated FAs (MUFAs). We then discovered that p73α1 directly inhibits Stearoyl-CoA Desaturase-1 (SCD1) expression, which converts saturated FAs to MUFAs. Finally, we found that p73α1-mediated suppression of *SCD1* inhibits cancer cell viability and leads to an increased ratio of saturated FAs to MUFAs.

## 2. Materials and Methods

### 2.1. Cell Culture and Cell Line Generation

H1299 cells (non-small cell lung carcinoma, ATCC; Manassas, VA, USA; Cat# CRL-5803) were cultured in DMEM (Gibco; Waltham, MA, USA; Cat# 12100-61) supplemented with 10% FBS (Gibco; Waltham, MA, USA; Cat# A4766801) and Antibiotic-Antimycotic solution (Gibco; Waltham, MA, USA; Cat# 15240-062). H1299 cell lines tested negative for mycoplasma and were used at passage 20 or lower. *E12*^−^^/^^−^ H1299 cell lines were generated as described previously [37].

### 2.2. Plasmid Generation

pSpCas9(BB)-2A-Puro expression vector was generated by the Zhang Lab [38] and purchased from Addgene (Watertown, MA, USA; Cat# 48139). A vector expressing a single guide RNA (sgRNA) that targeted *E12* was generated by annealing two 25-nt oligos and cloning the product into the pSpCas9(BB)-2A-Puro expression vector via BbsI. All primer sequences used to generate the corresponding expression vectors were listed in Table S1.

### 2.3. RNA Isolation and qPCR

Quick-RNA MiniPrep Kit (Zymo Research; Irvine, CA, USA; Cat# 11-327) was used to isolate RNA according to the manufacturer’s protocol. RNA was then used for cDNA synthesis using oligo dT (18) primer (Thermo Scientific; Waltham, MA, USA; Cat# FERSO123), random hexamer primer (Thermo Scientific; Waltham, MA, USA; Cat# SO142), dNTP (Cat# FERR0181), RiboLock RNase Inhibitor (Thermo Scientific; Waltham, MA, USA; Cat# EO0381), and RevertAid Reverse Transcriptase (Thermo Scientific; Waltham, MA, USA; Cat# EP0441) according to the manufacturer’s protocol. The cDNA was used for qPCR with PowerUp Sybr Green Master Mix (Applied Biosystems, Waltham, MA, USA; Cat# A25742) according to the manufacturer’s protocol. All primers used for qPCR were listed in Table S2.

### 2.4. Western Blot Analysis

Western blot analysis was performed as previously described [39]. Briefly, whole cell lysates were harvested with 1× SDS lysis buffer [62.5 mM Tris-HCl pH 6.5, 10% glycerol (Sigma; St. Louis, MO, USA; Cat# G5516-4L), 2% SDS, 0.71 M 2-mercaptoethanol (Acros Organics; Waltham, MA, USA; Cat# 125470010), and 0.15 mm bromophenol blue (Fisher Bioreagents; Waltham, MA, USA; Cat# BP115-25)] and boiled at 95 °C for 6 min. Proteins were separated on polyacrylamide gel [10% acrylamide/bis-acrylamide (Sigma; St. Louis, MO, USA; Cat# A3574-5L), 0.37 M Tris-HCl pH 8.8, 0.035% ammonium persulfate (VWR; Radnor, PA, USA; Cat# 0486-100G), and 4.6 M TEMED (Acros Organics; Waltham, MA, USA Cat# 138455000)], then transferred to 0.45 mM nitrocellulose membrane (Cytiva; Marlborough, MA, USA; Cat# 10600002). Membranes were probed overnight at 4 °C with the indicated primary antibodies: anti-CD92 (Santa Cruz Biotechnology; Dallas, TX, USA; Cat# 517098), anti-GAPDH (Cell Signaling Technology; Danvers, MA, USA; Cat# 14C10), anti-Vinculin (Cell Signaling Technology; Danvers, MA, USA; Cat# E1E9V), anti-CCTa (Cell Signaling Technology; Danvers, MA, USA; Cat# 6931S), anti-SMPD4 (Novus Biologicals; Littleton, CO, USA; Cat# NBP2-93253), anti-CEPT1 (Invitrogen; Waltham, MA, USA; Cat# PA5-23876) anti-SCD1 (Abcam; Cambridge, United Kingdom; Cat# ab236868). Membranes were then incubated at room temperature for 3 h with either anti-mouse (Bio Rad; Hercules, CA, USA; Cat# 1705047) or anti-rabbit (Bio Rad; Hercules, CA, USA; Cat# 1705046) HRP conjugated secondary antibodies. The proteins were visualized with WesternBright ECL HRP substrate (Advansta; San Jose, CA, USA; Cat# K-12043-D20). VisionWorks^®^LS software was used to analyze the images.

### 2.5. ChIP Assay

ChIP assay was performed as previously described [40]. Briefly, chromatin was cross-linked with 1% formaldehyde in phosphate-buffered saline (PBS) and added directly to the media. Cells were lysed in 2× modified RIPA buffer [0.1 M Tris-HCl, 2% NP-40 (USB; Waltham, MA, USA; Cat# 19628), 0.5% deoxycholic acid (Fisher Bioreagents; Waltham, MA, USA; Cat# BP349-100), 2 mm EDTA (Fisher Bioreagents; Waltham, MA, USA; Cat# BP120-1) with protease inhibitor cocktail (Thermo Scientific; Waltham, MA, USA; Cat# 78438). Chromatin lysates were sonicated to yield ~200–1000 base pair DNA fragments and immunoprecipitated with 1 mg of control anti-IgG normal rabbit (EMD Millipore; Burlington, MA, USA; Cat# NI01-100 mg) or anti-TAp73 (Bethyl Laboratories; Waltham, MA, USA; Cat# A300-126A) and captured with protein A/G magnetic agarose beads (Thermo Scientific; Waltham, MA, USA; Cat# 78609) at 4 °C overnight. The DNA-protein immunocomplexes were reverse cross-linked and purified using ChIP DNA Clean and Concentrator (Zymo Research; Irvine, CA, USA; Cat# 50-44-363). The DNA fragments were amplified with PCR using DreamTaq DNA polymerase (Thermo Scientific; Waltham, MA, USA; Cat# FEREP0713). The PCR program used for amplification was (1) 94 °C for 5 min, (2) 94 °C for 30 s, (3) 60 °C for 30 s, (4) 72 °C for 30 s, and (5) 72 °C for 10 min. Steps 2–4 were repeated for 32 cycles to amplify *GAPDH*, 36 cycles to amplify *SCD1*, or 38 cycles to amplify *CDKN1A*. Primers used for ChIP assay were listed in Table S2.

### 2.6. siRNA Knockdown

siRNA was purchased from Horizon Discovery Biosciences and resuspended in 5× siRNA Buffer (Thermo Scientific; Waltham, MA, USA; Cat# B-002000-UB-100) to a final concentration of 20 mM. 2 × 10^5^ cells were plated in a 6-well plate. After 24 h, cells were transfected using RNAi Max (Invitrogen; Waltham, MA, USA; Cat# 13778150) according to the manufacturer’s protocol with the appropriate siRNA at the indicated concentrations: scrambled siRNA (Scr) (5′-GGC CGA UUG UCA AAU AAU U-3′) (90 nM), si-p73α1 (5′- ACC UGG GGC CCG UGG UUU-3′) (70 nM), or si-SCD1 (5′-GAG AUA AGU UGG AGA CGA UdTdT-′3) (20 nM). After 48 h, cells were trypsinized (VWR; Radnor, PA, USA; Cat# 0458-250G) and either seeded for RNA or protein collection, or for cell viability assay.

### 2.7. Cell Viability Assay

5 × 10^3^ cells were plated in a 96-well plate. After 96 h, cell viability was measured using CellTiter-Glo 2.0 Cell Viability Assay kit (Promega; Madison, WI, USA; Cat# G9241) according to the manufacturer’s protocol. The assay was performed in triplicates to ensure proper statistical analyses.

### 2.8. Metabolomics and Lipidomics

Lipidomics and metabolomics analysis was performed at the UC Davis West Coast Metabolomics Center. Three million cells were collected into 2 mL Eppendorf tubes (Eppendorf; Hamburg, Germany; Cat# 4036-3352). Samples were randomized and extracted alongside one method blank and one bioreclamation sample per every 10 biological samples. Samples were extracted using the modified Matyash Extraction [41]. 225 µL of ice-cold methanol, with Avanti SPLASHone internal standards (Avanti Polar Lipids; Alabaster, AL, USA; Cat# 330707) was added to each sample. Cells were homogenized for 90 s in methanol with 2 mm stainless steel beads using an SPEX Geno/Grinder (SPEX SamplePrep; Metuchen, NJ, USA). 750 µL MTBE was added to the samples, vortex briefly, and then mixed on an orbital shaker for 5 min at 4 °C. Next, 188 µL water was added to the tubes, vortexed, and then centrifuged for 3 min at 16k RPM. The top organic layer was collected (two, 180 µL fractions) for lipidomics analysis, and the bottom polar fraction (two, 150 µL fractions) was collected for metabolomics. All fractions were dried under vacuum using a rotovap. Lipidomics analysis was performed by resuspending dried fractions in 100 µL Methanol: Toluene (9:1) and analyzed using liquid chromatography (LC) high-resolution mass spectrometry (Q Exactive HF MS/MS). LC conditions were carried out using a Vanquish Focused UHPLC, Waters Acquity UPLC CSH C18 columns (100 mm × 2.1 mm, 1.7 µm particle size) with mobile phase A consisting of 6:4 acetonitrile: water and mobile phase B consisting of 9:1 isopropanol: water, both containing 10 mM ammonium formate and 0.1% formic acid for positive ionization mode, and 10 mM ammonium formate for negative ionization mode analysis. 3 and 5 µLs of pooled samples were injected for positive and negative modes to equilibrate the column prior to analysis. Mobile phase gradient and mass spectrometry parameters were identical, as described previously [42]. Metabolomics was performed by GC-MS as previously described [43] and by high-resolution LC-MS/MS as described previously [44].

### 2.9. Statistical Analysis

Data are presented as mean ± SEM. Significant difference between two groups was assessed by one-tailed, unpaired Student’s *t*-test and comparisons between two or more groups were assessed by one-way ANOVA with Benjamini and Hochberg FDR post-test for multiple comparisons, when appropriate. Statistical analysis was performed with GraphPad Prism 9. Statistical parameters can be found in the figure legends. ChemRICH analysis was performed as previously described [45].

## 3. Results

### 3.1. Isoform Switch from p73α to p73α1 Alters the Metabolome in H1299 Cells

**Figure 1:**
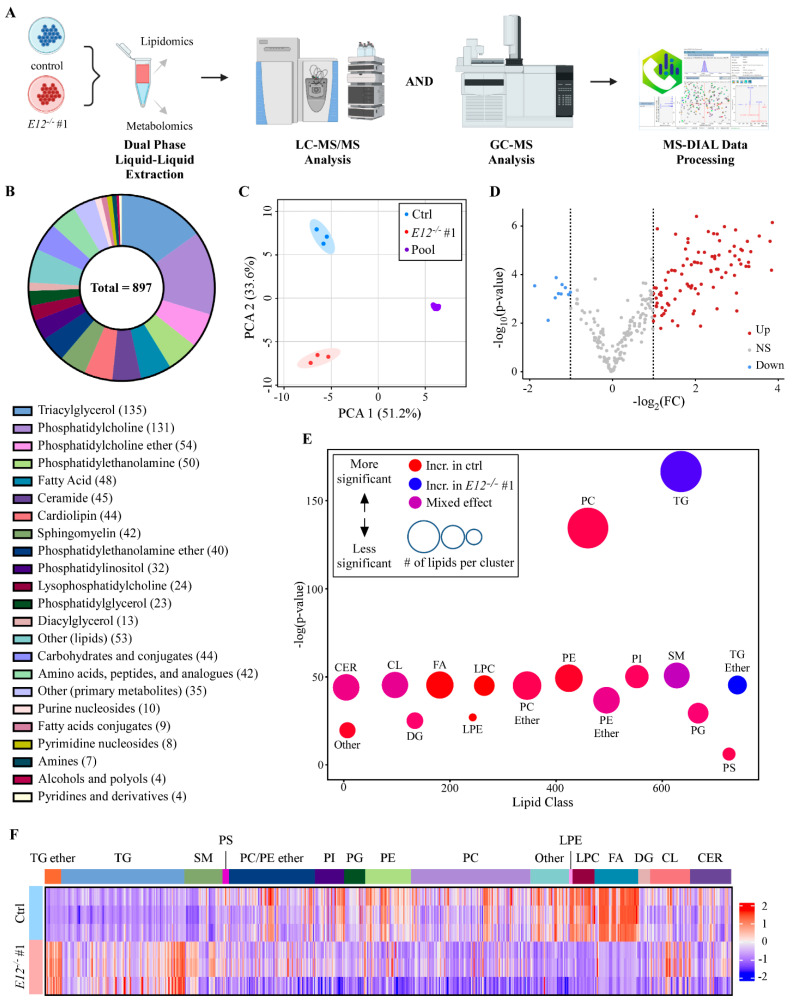
Lipidome overview in isogenic control and E12-KO H1299 cells. (**A**). Schematic of lipidomic and metabolomic analysis workflow in isogenic control and *E12*^−/−^ H1299 cells. (**B**). Class-level overview of metabolites annotated by LC-MS/MS and GC-MS analysis in isogenic control and *E12*^−/−^ H1299 cells. 897 individual metabolites were identified using accurate mass and in-silico libraries (top). Number of metabolites in each class are indicated in the parentheses (bottom). Classes with less than 10 identified lipids or metabolites were grouped together and denoted “Other (lipids)” or “Other (primary metabolites)”. (**C**). Principal component analysis (PCA) of isogenic control, *E12*^−/−^ H1299 cells and pooled quality control samples. (**D**). Volcano plot of isogenic control compared to *E12*^−/−^ H1299 cells. Up indicates lipids and metabolites higher in isogenic control cells. (**E**). Chemical similar enrichment analysis (ChemRICH) of all lipids identified in isogenic control and *E12*^−/−^ H1299 cells. Y-axis represents statistical significance assessed by Kolmogorov-Smirnov test and node size represents total number of lipids per cluster. Classes with less than 10 identified lipids were grouped together and denoted “Other”. TG: triacylglycerols; PC: phosphatidylcholines; CER: ceramides; CL: cardiolipins; FA: fatty acids; DG: diacylglycerols; LPE: lysophosphatidylethanolamines; LPC: lysophosphatidylcholines; PE: phosphatidylethanolamines; PI: phosphatidylinositols; PG: phosphatidylglycerols; PS: phosphatidylserines; SM: sphingomyelins. (**F**). Heatmap of all annotated lipids clustered by class in isogenic control and *E12*^−/−^ H1299 cells. Color intensity indicates z-score values of peak heights. CER: ceramides; CL: cardiolipins; DG: diacylglycerols; FA: fatty acids; LPC: lysophosphatidylcholines; LPE: lysophosphatidylethanolamines; PC: phosphatidylcholines; PE: phosphatidylethanolamines; PG: phosphatidylglycerols; PI: phosphatidylinositols; PS: phosphatidylserines; SM: sphingomyelins; TG: triacylglycerols.

To determine the role of p73α1 in regulating lipid metabolism, untargeted metabolomic and lipidomic analyses were performed in isogenic control and E12-KO clone #1 (*E12*^−/−^ #1) H1299 cell lines (Figure 1A). It is important to note that isogenic control cells express mostly p73α and a small amount of p73α1, and E12-KO cells express p73α1 and no p73α (Figure S1A) [37]. 734 lipids and 163 primary metabolites were identified through LC-MS/MS and GC-MS analysis, respectively. Triacylglycerols (TGs), PCs, ether-linked PCs, and PEs were among the classes with the highest lipid counts, and carbohydrates, amino acids, and peptides had the highest primary metabolite counts (Figure 1B). Principal Component Analysis showed the cell lines scattered in the first two principal components, with tight clustering of the pooled quality control samples, indicating excellent analytical precision. The first principal component explains 51% of the total observed biological variance, with the second principal component accounting for nearly 34% of the variance (Figure 1C). Total lipid content was found to be significantly lower in E12-KO cells compared to isogenic control cells (Figure S1B). Consistent with this, the volcano plot displayed a general trend of metabolites and lipids being downregulated in E12-KO cells compared to isogenic control cells (Figure 1D).

To identify class-level lipid changes associated with loss of *E12*, a chemical similarity enrichment analysis (ChemRICH) plot was generated. Using general class-level classifications, the analysis showed that PCs and TGs were the two most significantly altered classes; PCs were decreased, but TGs were increased in E12-KO cells, with a slightly mixed effect for both. The mixed effect for PCs indicated that many of these lipids were decreased in E12-KO cells, while some were increased, compared to isogenic control cells. Conversely, the mixed effect in TGs indicated that most TG lipids were increased in E12-KO cells, while some were decreased, compared to isogenic control cells (Figure 1E). To expand on the broad class-level analysis, a heat map detailing all annotated lipids was generated (Figure 1F). Loss of *E12* led to a considerable decrease in overall FAs compared to isogenic control cells (Figure 1F). Additionally, E12-KO cells exhibited decreased levels of PCs, LPCs, and PEs compared to isogenic control cells (Figure 1F). On the other hand, E12-KO cells elicited a substantial increase in TGs compared to isogenic control cells (Figure 1F). Carnitines and bis(monoacylglycerol)phosphates were significantly decreased in E12-KO cells compared to isogenic control cells, while ether-linked TGs exhibited the opposite trend (Figure S1C). Phosphatidylglycerols, cardiolipins, sphingomyelins, ceramides, and cholesterol esters were not altered between isogenic control and E12-KO cells (Figure S1D).

### 3.2. Loss of E12 Leads to Specific Lipidome Changes in H1299 Cells

**Figure 2:**
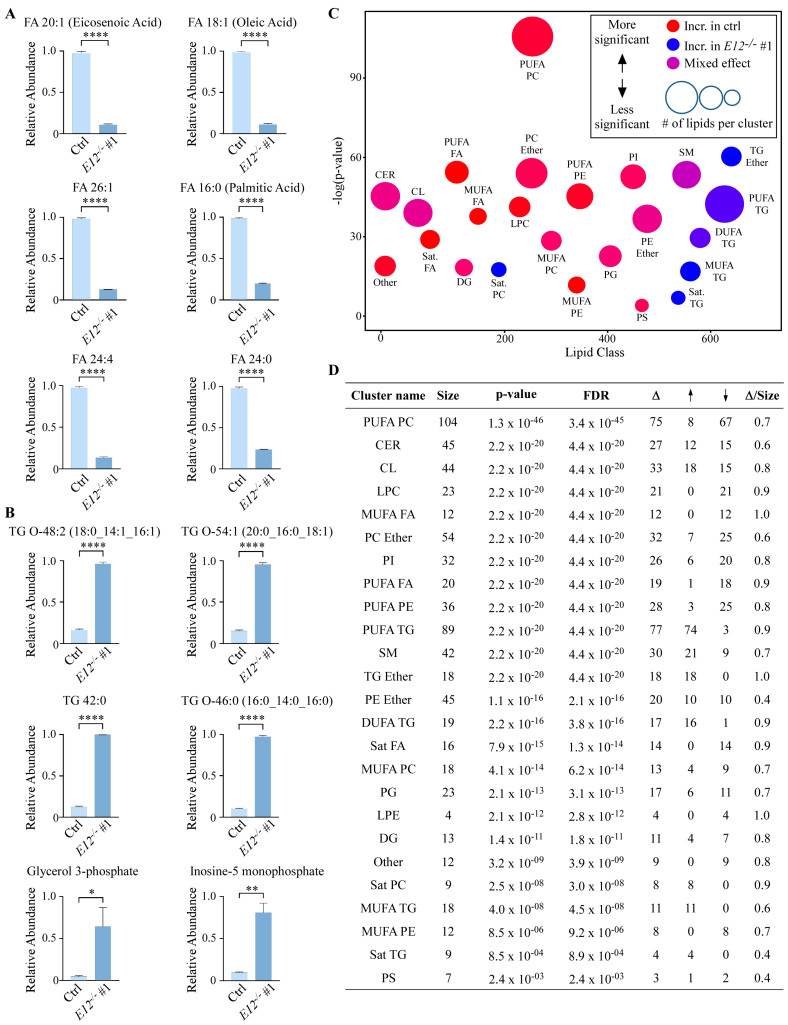
Loss of *E12* leads to lipidome changes in H1299 cells. A-B. (**A**) Top six downregulated and (**B**) upregulated metabolites in *E12*^−/−^ compared to isogenic control H1299 cells. TG-O indicates triacylglycerols with an ether bond. Statistical significance was determined using Student’s *t*-test. Data are presented as mean ± SEM. *n* = 3 independent experiments. * *p* < 0.05, ** *p* < 0.01, **** *p* < 0.0001. (**C**). ChemRICH of all lipids identified in isogenic control and *E12*^−/−^ H1299 cells. Y-axis represents statistical significance assessed by Kolmogorov-Smirnov test and node size represents total number of lipids per cluster. MUFA: mono-unsaturated fatty acids; DUFA: di-unsaturated fatty acids; PUFA: poly-unsaturated fatty acids; Sat: saturated. (**D**). Lipid clusters with significant alterations due to loss of *E12* determined by ChemRICH analysis. Δ indicates number of lipids altered in a cluster; ↑ indicates number of increased lipids in *E12*^−/−^ compared to isogenic control H1299 cells; ↓ indicates number of decreased lipids in *E12*^−/−^ compared to isogenic control H1299 cells; FDR indicates false discovery rate; Δ/size shows the proportion of significantly altered lipids within each class.

Lipidome changes between isogenic control and E12-KO cells were further investigated. The top six upregulated (Figure 2A) and downregulated (Figure 2B) compounds were analyzed to determine whether specific lipid classes or pathways were affected by the loss of *E12*. The top six downregulated metabolites were all FAs; three MUFAs (FA 18:1, FA 20:1, FA 26:1) all with fold changes of 0.10, two saturated FAs (FA 16:0, FA 24:0) with fold changes of 0.20 and 0.25, and one poly-unsaturated FA (PUFA) (FA 24:4) with a fold change of 0.14 (Figure 2A). TG species made up four of the top six upregulated metabolites, consisting of three ether-linked TGs (TG-O 48:2, TG-O 54:1, TG-O 46:0) with fold changes of 5, 5 and 10, respectively, and TG 42:0, with a fold change of 10 (Figure 2B). Two polar metabolites, glycerol 3-phosphate and inosine-5-monophosphate with fold changes of 14 and 10, were the other most upregulated metabolites. We hypothesized that the accumulation of glycerol 3-phosphate in E12-KO cells was a result of decreased glycerophospholipid levels, as shown in Figure 1F. Overall, these data indicate that loss of *E12* alters the abundance of certain lipid classes and precursors.

To better understand lipid metabolism alterations in E12-KO cells, the general class-level ChemRICH plot (Figure 1E) was delineated by saturation level (Figure 2C,D). Interestingly, the mixed effect seen in PCs and TGs in Figure 1E was not observed when the broad classes were stratified by saturation status. PCs containing PUFAs were significantly dysregulated (FDR = 3.4 × 10^45^), where 70% of all PCs containing PUFAs were altered (67 downregulated and 8 upregulated in E12-KO cells) (Figure 2D). On the other hand, PCs containing MUFAs had a slightly mixed effect, with 9 out of 13 being lower in E12-KO cells, and four out of 13 being higher in the E12-KO cells (Figure 2D). Interestingly, PCs containing saturated FAs were significantly upregulated (FDR = 2.5 × 10^8^) in E12-KO cells, with 90% being increased in E12-KO cells (Figure 2C,D). Overall, TGs of all saturation levels were increased in E12-KO cells compared to isogenic control cells (Figure 2C). All TG species containing saturated FAs and MUFAs were increased in E12-KO cells (Figure 2D). While over 90% of TGs containing di-unsaturated fatty acids (DUFAs) and PUFAs were increased in E12-KO cells, there were 4 individual species that were decreased in E12-KO cells (Figure 2D). Similar trends were seen for other lipid classes; however, several classes–ceramides, cardiolipins, sphingomyelins, and ether-linked PEs–showed a mixed effect (Figure 2D).

### 3.3. Kennedy Pathway Metabolites Are Altered upon Loss of E12 in H1299 Cells

**Figure 3:**
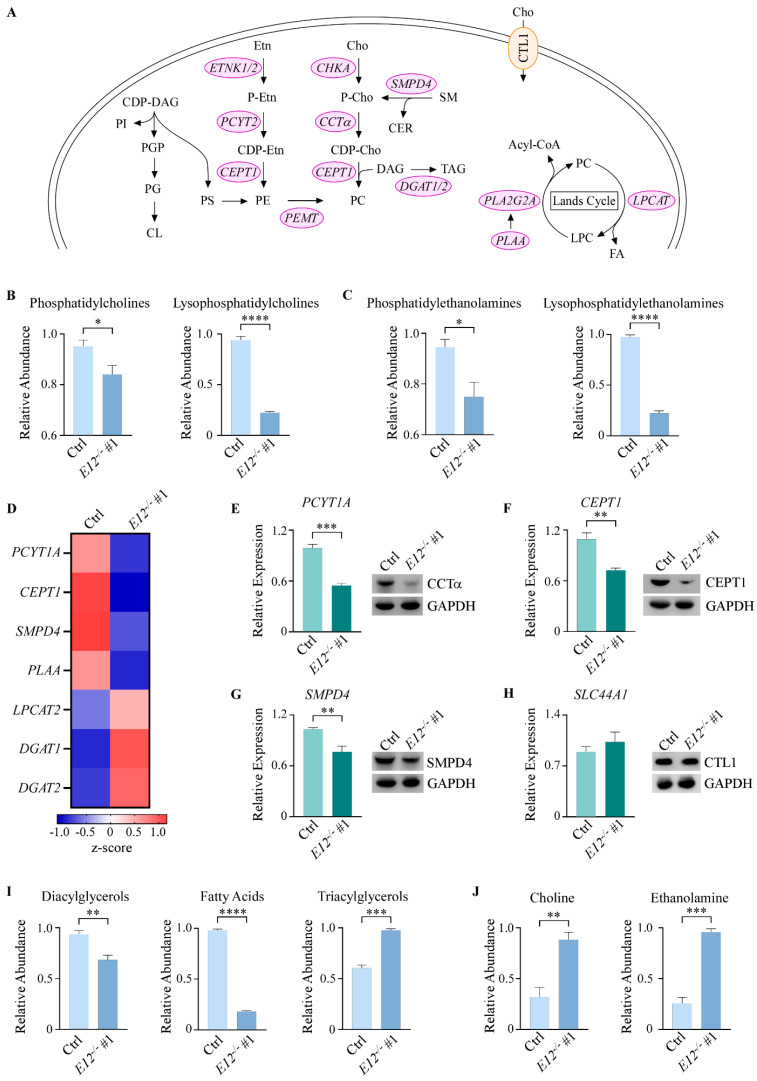
Kennedy pathway metabolites are altered upon knockout of *E12* in H1299 cells. (**A**). Schematic overview of the Kennedy pathway. CDP-DAG: CDP-diacylglycerol; PI: phosphatidylinositol; PG: phosphatidylglycerol; CL: cardiolipin; PS: phosphatidylserine; Cho: choline; P-cho: phosphocholine, CDP-cho: cytidine diphosphate-choline; Etn: ethanolamine, P-Etn: phosphoethanolamine; CDP-Etn: cytidine diphosphate-ethanolamine; SM: sphingomyelin; CER: ceramide; DAG: diacylglycerol; TAG: triacylglycerol; FA: fatty acid; CTL1: choline transporter-like protein 1. (**B**,**C**). Relative abundance of (**B**) phosphatidylcholines and lysophosphatidylcholines and (**C**) phosphatidylethanolamines and lysophosphatidylethanolamines in isogenic control and *E12*^−/−^ H1299 cells. Statistical significance was determined using Student’s *t*-test. (**D**). Heat map of differentially expressed genes identified by RNA-seq analysis in isogenic control and *E12*^−/−^ H1299 cell lines. Color density indicating z-score values was displayed below. (**E**–**H**). (Left) qPCR was used to quantify relative mRNA levels of *PCYT1A*, *CEPT1*, *SMPD4*, and *SLC44A1* in isogenic control and *E12*^−/−^ H1299 cell lines. All values were normalized to *HPRT1* and are presented as relative to isogenic control (light green). Statistical significance was determined using Student’s *t*-test. (Right) Western blot analysis was used to determine CCTa, CEPT1, SMPD4, CTL1, and GAPDH protein levels in isogenic control and *E12*^−/−^ H1299 cell lines. (**I**,**J**). Relative abundance of (**I**) diacylglycerols, fatty acids, and triacylglycerols, and (**E**) choline and ethanolamine in isogenic control and *E12*^−/−^ H1299 cells. Statistical significance was determined using Student’s *t*-test. For B-C and E-J. Data presented as mean ± SEM. *n* = 3 independent experiments. * *p* < 0.05, ** *p* < 0.01, *** *p* < 0.001, **** *p* < 0.0001.

As previously discussed, PCs are formed through the Kennedy pathway, the Lands Cycle, or the PEMT pathway (Figure 3A). ChemRICH analyses indicated that the Kennedy pathway was altered, so levels of PCs, LPCs, PEs, and lysophosphatidylethanolamines (LPEs) were further analyzed. Loss of *E12* led to a significant decrease in the four PC synthesis lipids compared to isogenic control cells (Figure 3B,C). Levels of the PEMT pathway methyl donor S-adenosylmethionine were more significantly increased in E12-KO cells, compared to the product of this reaction, S-adenosylhomocysteine (Figure S2A). These data suggest that the PEMT pathway might be inhibited by loss of *E12*, therefore leading to decreased PC production. We then postulated that loss of *E12* was altering the expression of genes involved in PC synthesis, thus explaining the observed lipid changes. To test this, previously generated RNA-seq data was analyzed and showed that several key genes involved in PC synthesis were differentially expressed in E12-KO cells compared to isogenic control cells (Figure 3D). mRNA and protein levels of several of these targets were then confirmed via qPCR and Western blot analysis. CCTα (encoded by *PCYT1A*) and CEPT1, two enzymes directly involved in PC synthesis, were significantly downregulated at the mRNA level and markedly decreased at the protein level in E12-KO cells compared to isogenic control cells (Figure 3E,F). Furthermore, SMPD4, which hydrolyzes sphingomyelin to ceramide and P-choline (Figure 3A), exhibited the same trend at both the mRNA and protein levels (Figure 3G). These data suggest that decreased PC levels in E12-KO cells are partly attributed to decreased expression of enzymes involved in PC synthesis.

DGs are necessary for the final step of PC synthesis and are also converted to TGs through the addition of acyl-CoA, which is catalyzed by diacylglycerol transferases (DGAT) [46] (Figure 3A). Levels of DGs and FAs were found to be significantly decreased in E12-KO cells, but TGs were significantly increased compared to isogenic control cells (Figure 3I). FAs are stored primarily as TGs [47], so increased TGs in E12-KO cells might explain why FAs are lower than in isogenic control cells. Moreover, the RNA-seq data showed that *DGAT1* and *DGAT2* were upregulated in E12-KO cells (Figure 3D), suggesting that DGs were shuttled more towards TG formation, rather than PC synthesis.

As previously mentioned, increased choline import is essential for tumor cells to maintain high levels of PCs. Due to this, it was determined whether decreased choline import was contributing to decreased PC levels in E12-KO cells. mRNA and protein levels of a major choline importer, CTL1 (encoded by *SLC44A1*), were analyzed and found to be unchanged in E12-KO cells compared to isogenic control cells (Figure 3H). Next, LC-MS/MS metabolomics was conducted to investigate intracellular choline and ethanolamine levels, and both were found to be significantly increased in E12-KO cells compared to isogenic control cells (Figure 3J). We hypothesize that the accumulation of choline and ethanolamine in E12-KO cells resulted from the decreased flux of these metabolites through the Kennedy pathway due to decreased expression of downstream genes, as discussed above. Altogether, these findings indicate that loss of *E12* leads to decreased PC levels due to decreased expression of enzymes directly or indirectly involved in PC/PE synthesis, and not due to decreased choline import.

### 3.4. Loss of E12 Alters PC Chain Length and Saturation in H1299 Cells

**Figure 4:**
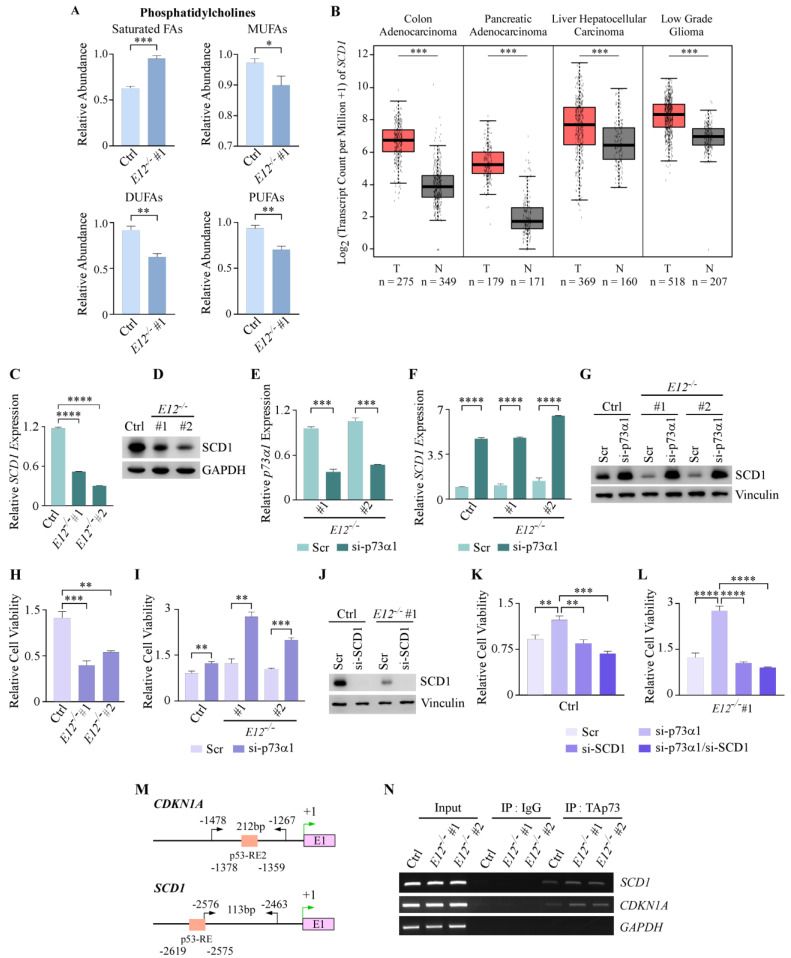
p73α1 suppresses cell viability by directly inhibiting *SCD1* expression. (**A**). Relative abundance of PC saturation level in isogenic control and *E12*^−/−^ H1299 cells. Statistical significance was determined using Student’s *t*-test. (**B**). *SCD1* transcript counts in the indicated tumors (data from TCGA) and the matched normal tissues (data from TCGA and GTEx) were analyzed via Gene Expression Profiling Interactive Analysis (GEPIA; http://gepia.cancer-pku.cn/index.html; accessed on 6 May 2022). T: tumor; N: matched normal tissue. (**C**). qPCR was used to quantify relative mRNA levels of *SCD1* in isogenic control and *E12*^−/−^ H1299 cell lines. All values were normalized to *HPRT1* and are presented as relative to isogenic control (light green). Statistical significance was determined using one-way ANOVA. (**D**). Western blot analysis was used to determine SCD1 and GAPDH protein levels in isogenic control and *E12*^−/−^ H1299 cell lines. (**E**). qPCR was used to quantify relative mRNA levels of *p73α1* in *E12*^−/−^ H1299 cells treated with scramble siRNA (Scr) or si-p73α1 for 48 h. All values were normalized to *HPRT1* and are presented as relative to each Scr-treated cell line. Statistical significance was determined using multiple Student’s *t*-tests comparing Scr to si-p73α1 treatment for each cell line. (**F**). Cells were treated as in (**C**) and qPCR was used to quantify relative mRNA levels of *SCD1*. All values were normalized to *HPRT1* and are presented as relative to each Scr-treated cell line. Statistical significance was determined using multiple Student’s *t*-tests comparing Scr to si-p73α1 treatment for each cell line. (**G**). Cells were treated as in (**C**) and Western blot analysis was used to determine SCD1 and Vinculin protein levels. (**H**). Cells were treated as in (**C**) and cell viability of the Scr-treated cell lines was determined using CellTiter-GLO. Cell viability for *E12*^−/−^ #1 and #2 (dark purple) were presented as relative to isogenic control cells (light purple). Statistical significance was determined using one-way ANOVA. (**I**). Cells were treated as in (**C**) and cell viability was determined using CellTiter-GLO. Cell viability for isogenic control cells treated with si-p73α1 was presented as relative to isogenic control Scr-treated cells. Cell viability for *E12*^−/−^ #1 cells treated with si-p73α1 was presented as relative to *E12*^−/−^ #1 Scr-treated cells. Cell viability for *E12*^−/−^ #2 cells treated with si-p73α1 was presented as relative to *E12*^−/−^ #2 Scr-treated cells. Statistical significance was determined using multiple Student’s *t*-tests comparing Scr to si-p73α1 treatment for each cell line. (**J**). Western blot analysis of SCD1 and Vinculin proteins in isogenic control and *E12*^−/−^ H1299 cell lines treated with Scr or si-SCD1 for 48 h. (**K**). Cell viability was determined using CellTiter-GLO in the isogenic control H1299 cell line treated with Scr, si-p73α1, si-SCD1, or si-p73α1 and si-SCD1 for 48 h. Cell viability for isogenic control cells treated with si-p73α1, si-SCD1, or si-p73α1 and si-SCD1 was presented as relative to isogenic control Scr-treated cells. Statistical significance was determined using one-way ANOVA. (**L**). Cell viability was determined using CellTiter-GLO in the *E12*^−/−^ H1299 cell line treated with Scr, si-p73α1, si-SCD1, or si-p73α1 and si-SCD1 for 48 h. Cell viability for *E12*^−/−^ cells treated with si-p73α1, si-SCD1, or si-p73α1 and si-SCD1 was presented as relative to *E12*^−/−^ Scr-treated cells. Statistical significance was determined using one-way ANOVA. (**M**). Diagram of the putative p53-Response Elements (p53-RE) (orange) in the promoter of *CDKN1A* and *SCD1*. Locations of the primers used to amplify the p53-RE in the promoters of *CDKN1A* and *SCD1* are indicated by the black arrows. Green arrows indicate transcription start site; E1 indicates exon 1. (**N**). ChIP analysis was performed with isogenic control and *E12*^−/−^ H1299 cells. Cell lysates were immunoprecipitated with anti-rabbit IgG or anti-TAp73 to bring down the DNA-protein complex. DNA fragments were visualized by PCR with primers for *SCD1*, *CDKN1A*, and *GAPDH* promoters. For (**A**,**C**,**E**,**F**,**H**,**I**,**K**,**L**). Data are presented as mean ± SEM. *n* = 3 independent experiments. * *p* < 0.05, ** *p* < 0.01, *** *p* < 0.001, **** *p* < 0.0001.

Multiple studies show that increased FA chain length and desaturation are associated with malignancy and tumorigenesis [48,49]. In this study, loss of *E12* did not alter levels of long-chain fatty acids (LCFAs), but levels of very long-chain fatty acids (VLCFAs) were significantly decreased compared to isogenic control cells (Figure S2B). Consistent with the data in Figure 2D, a deeper analysis of PC saturation showed that, when compared to isogenic control cells, E12-KO cells had significantly higher levels of PCs containing saturated FAs and lower levels of PCs containing MUFAs, DUFAs, and PUFAs (Figure 4A). These findings suggest that loss of *E12* leads to dysregulation of PC chain length and saturation.

### 3.5. p73α1 Suppresses Cancer Cell Viability by Directly Inhibiting SCD1 Expression in H1299 Cells

The finding that loss of *E12* led to increased saturated FAs and decreased MUFAs was further explored given the importance of MUFAs in tumorigenesis. There are three human desaturase enzymes (Δ5, Δ6 and Δ9) that are responsible for the formation of MUFAs, DUFAs, and PUFAs [50,51]. The rate-limiting step is the conversion of saturated FAs to MUFAs, which is catalyzed by Δ9 desaturase, or SCD1 [52]. SCD1 primarily catalyzes the formation of palmitoleic acid (FA 16:1) and oleic acid (FA 18:1) from palmitic acid (FA 16:0) and stearic acid (18:0), respectively [53]. Oleic acid and palmitoleic acid are the most abundant intracellular MUFAs and are necessary for the production of many lipids [54]. Interestingly, increased SCD1 expression is highly implicated in a variety of cancer types because of the tumorigenic properties of MUFAs [55,56,57]. Analysis of The Cancer Genome Atlas and the Genotype-Tissue Expression databases showed that *SCD1* transcript levels were significantly increased in 17 out of 31 tumor types compared to the matched normal tissues (Figure 4B; Figure S3A,B). Previously, p53 was identified as a direct suppressor of *SCD1* expression [58], and it is known that p73 can bind to p53-response elements to regulate target gene expression [59]. Given this, we hypothesized that p73α1 directly inhibits *SCD1* expression, therefore suppressing cancer cell proliferation.

To test this, SCD1 mRNA and protein levels were analyzed in isogenic control and E12-KO H1299 cell lines. mRNA levels were significantly decreased, and protein levels were considerably decreased in E12-KO cells compared to isogenic control cells (Figure 4C,D). Next, p73α1-specific siRNA was used to determine whether p73α1 was responsible for the observed decrease in SCD1 mRNA and protein levels (Figure 4E). Indeed, the data revealed that knockdown of p73α1 in both isogenic control and E12-KO cells led to a significant increase in *SCD1* mRNA levels, and a consistent increase at the protein level (Figure 4F,G). We then wanted to determine whether p73α1-mediated suppression of *SCD1* expression was contributing to the previously described tumor suppressive effects of p73α1. First, it was reiterated that loss of *E12* leads to decreased cell viability (Figure 4H), and that p73α1 was responsible for the growth-suppressive effects (Figure 4I). Next, siRNA targeting p73α1 and SCD1 (Figure 4J) was used to determine whether p73α1-mediated suppression of *SCD1* inhibits cancer cell proliferation. In both isogenic control and E12-KO cells, knockdown of *SCD1*, alone or with p73α1, led to a significant decrease in cell viability compared to knockdown of only p73α1 (Figure 4K,L). Moreover, knockdown of p73α1 and *SCD1* did not elicit a significant difference in cell viability compared to knockdown of *SCD1* alone (Figure 4K,L), confirming that p73α1-mediated suppression of *SCD1* contributes to the decreased cell viability in E12-KO cells.

To determine whether p73α1 was directly inhibiting *SCD1* expression, a chromatin immunoprecipitation (ChIP) assay was performed in isogenic control and E12-KO cell lines (Figure 4M). The data showed that DNA fragments containing the putative p53-response element in the *SCD1* promoter were detected following immunoprecipitation with TAp73 antibody in both isogenic control and E12-KO cell lines (Figure 4N). Taken together, these data identify SCD1 as a direct target of p73α1 and show that p73α1-mediated suppression of *SCD1* expression contributes to the tumor suppressive effects of p73α1.

### 3.6. p73α1-Mediated Suppression of SCD1 Leads to an Increased Ratio of Stearic Acid to Oleic Acid in H1299 Cells

**Figure 5:**
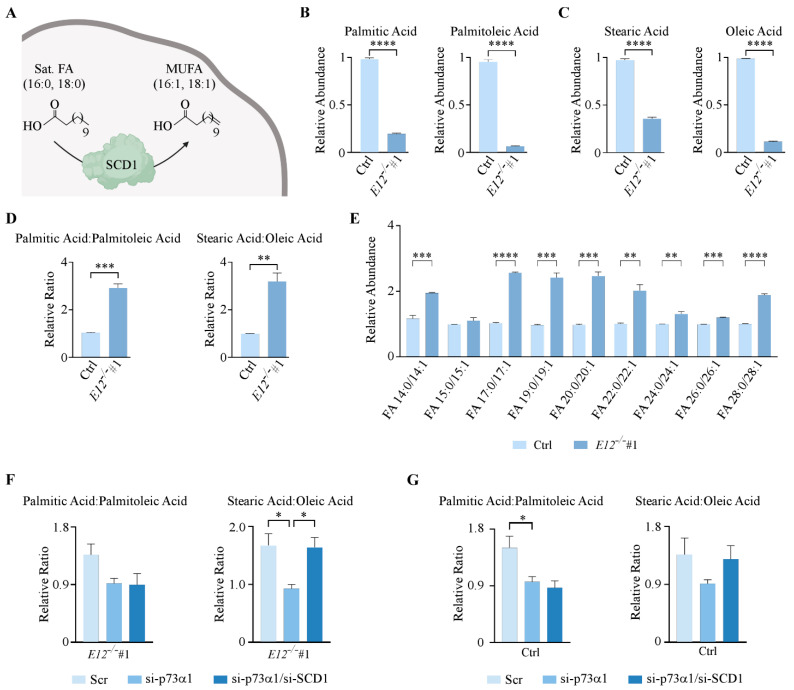
p73α1-mediated suppression of *SCD1* leads to an increased ratio of saturated FAs to MUFAs. (**A**). Schematic representation of SCD1 enzymatic activity. (**B**,**C**). Relative abundance of (**B**) palmitic acid and palmitoleic acid and (**C**) stearic acid and oleic acid in isogenic control and *E12*^−/−^ H1299 cells. Statistical significance was determined using Student’s *t*-test. (**D**). Ratio of (left) palmitic acid to palmitoleic acid and (right) stearic acid to oleic acid in isogenic control and *E12*^−/−^ H1299 cells. Statistical significance was determined using Student’s *t*-test. (**E**). Ratio of saturated FAs to MUFAs for the indicated FA chain length in isogenic control (light blue) and *E12*^−/−^ (dark blue) H1299 cells. Statistical significance was determined using multiple Student’s *t*-tests comparing isogenic control and *E12*^−/−^ cell lines for each saturated/unsaturated FA pair. (**F**). Ratio of (left) palmitic acid to palmitoleic acid and (right) stearic acid to oleic acid in *E12*^−/−^ H1299 cells following treatment with Scr, si-SCD1, or si-SCD1 and si-p73α1 for 48 h. Statistical significance was determined using one-way ANOVA. (**G**). Ratio of (left) palmitic acid to palmitoleic acid and (right) stearic acid to oleic acid in isogenic control H1299 cells treated with Scr, sip73α1, or sip73α1 and siSCD1 for 48 h. Statistical significance was determined using one-way ANOVA. For B-G. Data are presented as mean ± SEM. *n* = 3 independent experiments. * *p* < 0.05, ** *p* < 0.01, *** *p* < 0.001, **** *p* < 0.0001.

As previously mentioned, palmitic acid and stearic acid are the major substrates for SCD1, leading to the formation of palmitoleic acid and oleic acid, respectively (Figure 5A). Due to the findings that p73α1 directly suppresses *SCD1* expression, it was determined whether this affected the levels of these four lipids. E12-KO cells had significantly decreased levels of these four FAs compared to isogenic control cells (Figure 5B,C). Next, the ratios of saturated FAs to MUFAs were analyzed because a change in SCD1 expression and/or activity would alter these ratios. Indeed, the data showed that the ratios of palmitic acid to palmitoleic acid and stearic acid to oleic acid were significantly increased in E12-KO cells compared to isogenic control cells (Figure 5D). Additionally, the ratios of saturated FAs to MUFAs of varying chain lengths were analyzed, and 8 out of 9 were significantly increased in E12-KO cells compared to isogenic control cells (Figure 5E).

To confirm that p73α1-mediated suppression of *SCD1* expression leads to an increased ratio of saturated FAs to MUFAs, lipidomic analysis was performed following knockdown of p73α1 or p73α1 and SCD1. The ratio of stearic acid to oleic acid was significantly decreased after knockdown of p73α1, with a similar, albeit not significant, trend seen in the ratio of palmitic acid to palmitoleic acid in E12-KO cells (Figure 5F). Following knockdown of both p73α1 and SCD1 in E12-KO cells, there was a significant increase in the ratio of stearic acid to oleic acid, but not in the ratio of palmitic acid to palmitoleic acid (Figure 5F). A similar trend for both ratios was found in the isogenic control cells (Figure 5G). Taken together, these data identify p73α1 as a transcriptional repressor of *SCD1*, which leads to an increased ratio of stearic to oleic acid.

## 4. Discussion

SCD1 promotes tumorigenesis through a decreased ratio of saturated FAs to MUFAs [55,56,57]. Phospholipids containing MUFAs are less susceptible to lipid peroxidation than saturated FAs and PUFAs, therefore conferring resistance to ferroptosis [60,61]. Moreover, mass-spectrometry-based imaging showed that PCs containing MUFAs were more abundant in cancerous tissues compared to matched normal tissues [62]. Conversely, the accumulation of saturated FAs causes lipotoxicity, and in most cases cell death, by promoting endoplasmic reticulum stress [63]. These findings support a mechanism for how suppression of *SCD1* expression via p73α1 leads to decreased cancer cell viability in E12-KO cells. In the present study, we found that p73α1-knockdown decreased the ratio of stearic acid to oleic acid, while knockdown of both p73α1 and SCD1 reversed these effects. Interestingly, the reversal following concurrent knockdown was not observed in the ratio of palmitic acid to palmitoleic acid. One report noted that some cancer cells are able to utilize Δ6 desaturase (FADS2) to produce cis-6-C16:1 (FA 16:1; sapienate) [64], which differs from palmitoleic acid in the location of the double bond. By itself, FADS2 might be able to compensate for the loss of SCD1, which is why the ratio of FA 16:0 to FA 16:1 is not increased following SCD1 knockdown. However, we were unable to give detailed chemical analyses of the possible FA 16:1-containing isomers because current metabolomics methods cannot determine the location of double bonds in complex lipids. Such detailed analyses may be possible in the future by adding ultraviolet photodissociation mass spectrometry [65], chemical derivatization such as ozonolysis [66], or electron-activated dissociation mass spectrometry [67].

Cancer cells are able to increase uptake and/or biosynthesis of FAs, allowing for increased energy for various cellular processes, such as growth and proliferation [68]. In this study, we not only found that loss of *E12* altered the ration of saturated FAs to MUFAs, but we also showed that, overall, FA abundance was decreased upon E12-KO. We hypothesize that decreased FA abundance in E12-KO cells could also be contributing to the observed decrease in cell viability. Moreover, decreased abundance of FAs could lead to decreased mitochondrial FA oxidation, which has been shown to be increased in tumorigenic cells [68]. As such, it would be interesting to explore the relationship between cancer cell viability and FA abundance and oxidation in E12-KO cells in future work.

In addition to the actual biochemical differences that we observed, we are also able to interpret these changes with respect to the organelles that may be most likely involved. The peroxisome is a key organelle involved in lipid metabolism, immunometabolism, and cellular redox balance. It is the only organelle that catabolizes VLCFAs and branched-chain FAs, and converts FAs and alcohols to ether-linked lipids. Our data showed that the loss of *E12* resulted in increased catabolism of VLCFAs, indicating alterations to peroxisomal activity. Additional lines of evidence to support this notion are increased levels of ether-linked TG lipids in E12-KO cells compared to isogenic control cells. Interestingly, phospho-ether lipids did not show differences in abundance, in contrast with ether-linked TG lipids. Such a phenomenon has not been reported before. Many studies have shown that peroxisomal alterations contribute to cancer and inflammation. A link of peroxisomal activity to cancer cell autophagy was previously shown by the impaired ability of CD8 + T-cells to kill malignant cells that were associated with an accumulation of LCFAs and VLCFAs within the tumor microenvironment [69]. Ether-linked lipids have also been shown to be elevated in various tumors compared to control tissues, and show a linear relationship with metastatic spread in breast, prostate, and lung cancers [68,70,71]. We previously reported that *E12^+/−^*mice had increased immune cell infiltration and inflammation [37], which may be explained by peroxisomal-derived inflammatory cytokines and metabolites (prostaglandins, leukotrienes, thromboxanes) and immune cell activation through redox homeostasis. Future research will examine the role of the peroxisome in relationship to p73α1 to better understand tumor microenvironment and metabolism, and overall cancer cell phenotypes.

## 5. Conclusions

In this study, we identified a role for p73α1 in lipid metabolism through direct regulation of SCD1, which alters the ratio of saturated FAs to MUFAs and decreases cancer cell viability. Taken together, our data indicate that p73 has a critical role in regulating the metabolome and lipidome, which may contribute to oncogenesis, redox balance, and immunometabolic signaling. As such, it would be of great interest to further investigate how the various p73 isoforms alter biochemical pathways, thus influencing the tumor microenvironment and cancer metabolism.

## Data Availability

Available upon request.

## Supplementary Materials

The following supporting information can be downloaded at: https://www.mdpi.com/article/10.3390/cells11162516/s1, Figure S1: Loss of *E12* alters the lipidome in H1299 cells; Figure S2: Metabolites involved in PC synthesis are altered upon loss of *E12*; Figure S3: *SCD1* transcript counts are increased in certain cancer types; Table S1: Primers used to generate expression vectors; Table S2: Primers used for qPCR and ChIP.

## References

[1] Tocher D.R. (1995). Chapter 6 Glycerophospholipid metabolism. Biochemistry and Molecular Biology of Fishes.

[2] Kanno K., Wu M.K., Scapa E.F., Roderick S.L., Cohen D.E. (2007). Structure and function of phosphatidylcholine transfer protein (PC-TP)/StarD2. Biochim. Biophys. Acta-Mol. Cell Biol. Lipids.

[3] Gibellini F., Smith T.K. (2010). The Kennedy pathway-de novo synthesis of phosphatidylethanolamine and phosphatidylcholine. IUBMB Life.

[4] Moessinger C., Klizaite K., Steinhagen A., Philippou-Massier J., Shevchenko A., Hoch M., Ejsing C.S., Thiele C. (2014). Two different pathways of phosphatidylcholine synthesis, the Kennedy Pathway and the Lands Cycle, differentially regulate cellular triacylglycerol storage. BMC Cell Biol..

[5] Watkins S.M., Zhu X., Zeisel S.H. (2003). Phosphatidylethanolamine-N-methyltransferase Activity and Dietary Choline Regulate Liver-Plasma Lipid Flux and Essential Fatty Acid Metabolism in Mice. J. Nutr..

[6] Michel V., Yuan Z., Ramsubir S., Bakovic M. (2006). Choline transport for phospholipid synthesis. Exp. Biol. Med..

[7] Robichaud P.P., Surette M.E. (2015). Polyunsaturated fatty acid-phospholipid remodeling and inflammation. Curr. Opin. Endocrinol. Diabetes Obes..

[8] Vance D.E. (2014). Phospholipid methylation in mammals: From biochemistry to physiological function. Biochim. Biophys. Acta-Biomembr..

[9] Harayama T., Eto M., Shindou H., Kita Y., Otsubo E., Hishikawa D., Ishii S., Sakimura K., Mishina M., Shimizu T. (2014). Lysophospholipid acyltransferases mediate phosphatidylcholine diversification to achieve the physical properties required in vivo. Cell Metab..

[10] Cole L.K., Vance J.E., Vance D.E. (2012). Phosphatidylcholine biosynthesis and lipoprotein metabolism. Biochim. Biophys. Acta-Mol. Cell Biol. Lipids.

[11] Hanahan D., Weinberg R.A. (2011). Hallmarks of cancer: The next generation. Cell.

[12] Snaebjornsson M.T., Janaki-Raman S., Schulze A. (2020). Greasing the Wheels of the Cancer Machine: The Role of Lipid Metabolism in Cancer. Cell Metab..

[13] Saito T., Kuma A., Sugiura Y., Ichimura Y., Obata M., Kitamura H., Okuda S., Lee H.C., Ikeda K., Kanegae Y. (2019). Autophagy regulates lipid metabolism through selective turnover of NCoR1. Nat. Commun..

[14] Jain M., Nilsson R., Sharma S., Madhusudhan N., Kitami T., Souza A.L., Kafri R., Kirschner M.W., Clish C.B., Mootha V.K. (2012). Metabolite profiling identifies a key role for glycine in rapid cancer cell proliferation. Science.

[15] Daly P.F., Lyon R.C., Faustino P.J., Cohen J.S. (1987). Phospholipid metabolism in cancer cells monitored by 31P NMR spectroscopy. J. Biol. Chem..

[16] Glunde K., Bhujwalla Z.M., Ronen S.M. (2011). Choline metabolism in malignant transformation. Nat. Rev. Cancer.

[17] Zheng Y., Rodrik V., Toschi A., Shi M., Hui L., Shen Y., Foster D.A. (2006). Phospholipase D Couples Survival and Migration Signals in Stress Response of Human Cancer Cells. J. Biol. Chem..

[18] Gomez-Cambronero J. (2014). Phosphatidic acid, phospholipase D and tumorigenesis. Adv. Biol. Regul..

[19] Han H., Qi R., Zhou J.J., Ta A.P., Yang B., Nakaoka H.J., Seo G., Guan K.L., Luo R., Wang W. (2018). Regulation of the Hippo Pathway by Phosphatidic Acid-Mediated Lipid-Protein Interaction. Mol. Cell.

[20] Foster D.A. (2009). Phosphatidic acid signaling to mTOR: Signals for the survival of human cancer cells. Biochim. Biophys. Acta-Mol. Cell Biol. Lipids.

[21] Wang B., Wu L., Chen J., Dong L., Chen C., Wen Z., Hu J., Fleming I., Wang D.W. (2021). Metabolism pathways of arachidonic acids: Mechanisms and potential therapeutic targets. Signal Transduct. Target. Ther..

[22] Kaghad M., Bonnet H., Yang A., Creancier L., Biscan J.C., Valent A., Minty A., Chalon P., Lelias J.M., Dumont X. (1997). Monoallelically expressed gene related to p53 at 1p36, a region frequently deleted in neuroblastoma and other human cancers. Cell.

[23] Jost C.A., Marin M.C., Kaelin W.G. (1997). P73 is a human p53-related protein that can induce apoptosis. Nature.

[24] Arrowsmith C.H. (1999). Structure and function in the p53 family. Cell Death Differ..

[25] Tomasini R., Tsuchihara K., Wilhelm M., Fujitani M., Rufini A., Cheung C.C., Khan F., Itie-Youten A., Wakeham A., Tsao M.S. (2008). TAp73 knockout shows genomic instability with infertility and tumor suppressor functions. Genes Dev..

[26] Zhu J., Jiang J., Zhou W., Chen X. (1998). The Potential Tumor Suppressor p73 Differentially Regulates Cellular p53 Target Genes. Cancer Res..

[27] Melino G., Bernassola F., Ranalli M., Yee K., Zong W.X., Corazzari M., Knight R.A., Green D.R., Thompson C., Vousden K.H. (2004). P73 Induces Apoptosis via PUMA Transactivation and Bax Mitochondrial Translocation. J. Biol. Chem..

[28] Vernole P., Neale M.H., Barcaroli D., Munarriz E., Knight R.A., Tomasini R., Mak T.W., Melino G., de Laurenzi V. (2009). TAp73α binds the kinetochore proteins Bub1 and Bub3 resulting in polyploidy. Cell Cycle.

[29] Yang A., Walker N., Bronson R., Kaghad M., Oosterwegel M., Bonnin J., Vagner C., Bonnet H., Dikkes P., Sharpe A. (2000). P73-deficient mice have neurological, pheromonal and inflammatory defects but lack spontaneous tumours. Nature.

[30] Wilhelm M.T., Rufini A., Wetzel M.K., Tsuchihara K., Inoue S., Tomasini R., Itie-Youten A., Wakeham A., Arsenian-Henriksson M., Melino G. (2010). Isoform-specific p73 knockout mice reveal a novel role for ΔNp73 in the DNA damage response pathway. Genes Dev..

[31] Zaika A.I., Slade N., Erster S.H., Sansome C., Joseph T.W., Pearl M., Chalas E., Moll U.M. (2002). δNp73, a dominant-negative inhibitor of wild-type p53 and TAp73, is up-regulated in human tumors. J. Exp. Med..

[32] Steder M., Alla V., Meier C., Spitschak A., Pahnke J., Fürst K., Kowtharapu B.S., Engelmann D., Petigk J., Egberts F. (2013). DNp73 Exerts Function in Metastasis Initiation by Disconnecting the Inhibitory Role of EPLIN on IGF1R-AKT/STAT3 Signaling. Cancer Cell.

[33] De Laurenzi V., Costanzo A., Barcaroli D., Terrinoni A., Falco M., Annicchiarico-petruzzelli M., Levrero M., Melino G. (1998). Two New p73 Splice Variants with Different Transcriptional Activity. J. Exp. Med..

[34] de Laurenzi V., Catani M.V., Terrinoni A., Corazzari M., Melino G., Constanzo A., Levrero M., Knight R.A. (1999). Additional complexity in p73: Induction by mitogens in lymphoid cells and identification of two new splicing variants ε and ζ. Cell Death Differ..

[35] Rufini A., Agostini M., Grespi F., Tomasini R., Sayan B.S., Niklison-Chirou M.V., Conforti F., Velletri T., Mastino A., Mak T.W. (2011). P73 in cancer. Genes Cancer.

[36] Grespi F., Amelio I., Tucci P., Annicchiarico-Petruzzelli M., Melino G. (2012). Tissue-specific expression of p73 C-terminal isoforms in mice. Cell Cycle.

[37] Laubach K.N., Yan W., Kong X., Sun W., Chen M., Zhang J., Chen X. (2022). p73α1, a p73 C-terminal isoform, regulates tumor suppression and the inflammatory response via Notch1. Proc. Natl. Acad. Sci. USA.

[38] Ran F.A., Hsu P.D., Wright J., Agarwala V., Scott D.A., Zhang F. (2013). Genome engineering using the CRISPR-Cas9 system. Nat. Protoc..

[39] Chen X., Bargonetti J., Prives C. (1995). p53, through p21 (WAF1/CIP1), Induces Cyclin D1 Synthesis. Cancer Res..

[40] Liu G., Xia T., Chen X. (2003). The Activation Domains, the Proline-rich Domain, and the C-terminal Basic Domain in p53 Are Necessary for Acetylation of Histones on the Proximal p21 Promoter and Interaction with p300/CREB-binding Protein. J. Biol. Chem..

[41] Matyash V., Liebisch G., Kurzchalia T.V., Shevchenko A., Schwudke D. (2008). Lipid extraction by methyl-tert-butyl ether for high-throughput lipidomics. J. Lipid Res..

[42] Folz J., Oh Y.T., Blaženović I., Richey J., Fiehn O., Youn J.H. (2019). Interaction of Gut Microbiota and High-Sodium, Low-Potassium Diet in Altering Plasma Triglyceride Profiles Revealed by Lipidomics Analysis. Mol. Nutr. Food Res..

[43] Showalter M.R., Nonnecke E.B., Linderholm A.L., Cajka T., Sa M.R., Lönnerdal B., Kenyon N.J., Fiehn O. (2018). Obesogenic diets alter metabolism in mice. PLoS ONE.

[44] Rabow Z., Morningstar T., Showalter M., Heil H., Thongphanh K., Fan S., Chan J., Martínez-Cerdeño V., Berman R., Zagzag D. (2021). Exposure to DMSO during infancy alters neurochemistry, social interactions, and brain morphology in long-evans rats. Brain Behav..

[45] Barupal D.K., Fiehn O. (2017). Chemical Similarity Enrichment Analysis (ChemRICH) as alternative to biochemical pathway mapping for metabolomic datasets. Sci. Rep..

[46] Eichmann T.O., Lass A. (2015). DAG tales: The multiple faces of diacylglycerol—Stereochemistry, metabolism, and signaling. Cell Mol. Life Sci..

[47] Koundouros N., Poulogiannis G. (2019). Reprogramming of fatty acid metabolism in cancer. Br. J. Cancer.

[48] Chen M., Huang J. (2019). The expanded role of fatty acid metabolism in cancer: New aspects and targets. Precis. Clin. Med..

[49] Nagarajan S.R., Butler L.M., Hoy A.J. (2021). The diversity and breadth of cancer cell fatty acid metabolism. Cancer Metab..

[50] Paton C.M., Ntambi J.M. (2009). Biochemical and physiological function of stearoyl-CoA desaturase. Am. J. Physiol.-Endocrinol. Metab..

[51] Nakamura M.T., Nara T.Y. (2004). Structure, Function, and Dietary Regulation of Δ6, Δ5, and Δ9 Desaturases. Annu. Rev. Nutr..

[52] Ntambi J.M. (1995). The regulation of stearoyl-CoA desaturase (SCD). Prog. Lipid Res..

[53] Enoch H.G., Catala A., Strittmatter P. (1976). Mechanism of rat liver microsomal stearyl-CoA desaturase. Studies of the substrate specificity, enzyme-substrate interactions, and the function of lipid. J. Biol. Chem..

[54] Burlingame B., Nishida C., Uauy R., Weisell R. (2009). Fats and Fatty Acids in Human Nutrition: Introduction. Ann. Nutr. Metab..

[55] Roongta U.V., Pabalan J.G., Wang X., Ryseck R.P., Fargnoli J., Henley B.J., Yang W.P., Zhu J., Madireddi M.T., Lawrence R.M. (2011). Cancer cell dependence on unsaturated fatty acids implicates stearoyl-CoA desaturase as a target for cancer therapy. Mol. Cancer Res..

[56] Igal R.A. (2010). Stearoyl-coa desaturase-1: A novel key player in the mechanisms of cell proliferation, programmed cell death and transformation to cancer. Carcinogenesis.

[57] von Roemeling C.A., Marlow L.A., Radisky D.C., Rohl A., Larsen H.E., Wei J., Sasinowska H., Zhu H., Drake R., Sasinowski M. (2014). Functional genomics identifies novel genes essential for clear cell renal cell carcinoma tumor cell proliferation and migration. Oncotarget.

[58] Kirschner K., Samarajiwa S.A., Cairns J.M., Menon S., Pérez-Mancera P.A., Tomimatsu K., Bermejo-Rodriguez C., Ito Y., Chandra T., Narita M. (2015). Phenotype Specific Analyses Reveal Distinct Regulatory Mechanism for Chronically Activated p53. PLoS Genet..

[59] Harms K., Nozell S., Chen X. (2004). The common and distinct target genes of the p53 family transcription factors. C. Cell. Mol. Life Sci..

[60] Luis G., Godfroid A., Nishiumi S., Cimino J., Blacher S., Maquoi E., Wery C., Collignon A., Longuespée R., Montero-Ruiz L. (2021). Tumor resistance to ferroptosis driven by Stearoyl-CoA Desaturase-1 (SCD1) in cancer cells and Fatty Acid Biding Protein-4 (FABP4) in tumor microenvironment promote tumor recurrence. Redox Biol..

[61] Scott J.S., Nassar Z.D., Swinnen J.V., Butler L.M. (2022). Monounsaturated Fatty Acids: Key Regulators of Cell Viability and Intracellular Signaling in Cancer. Mol. Cancer Res..

[62] Guo S., Wang Y., Zhou D., Li Z. (2014). Significantly increased monounsaturated lipids relative to polyunsaturated lipids in six types of cancer microenvironment are observed by mass spectrometry imaging. Sci. Rep..

[63] Ackerman D., Simon M.C. (2014). Hypoxia, lipids, and cancer: Surviving the harsh tumor microenvironment. Trends Cell Biol..

[64] Vriens K., Christen S., Parik S., Broekaert D., Yoshinaga K., Talebi A., Dehairs J., Escalona-Noguero C., Schmieder R., Cornfield T. (2019). Evidence for an alternative fatty acid desaturation pathway increasing cancer plasticity. Nature.

[65] Williams P.E., Klein D.R., Greer S.M., Brodbelt J.S. (2017). Pinpointing Double Bond and sn-Positions in Glycerophospholipids via Hybrid 193 nm Ultraviolet Photodissociation (UVPD) Mass Spectrometry. J. Am. Chem. Soc..

[66] Harris R.A., May J.C., Stinson C.A., Xia Y., McLean J.A. (2018). Determining Double Bond Position in Lipids Using Online Ozonolysis Coupled to Liquid Chromatography and Ion Mobility-Mass Spectrometry. Anal. Chem..

[67] Baba T., Ryumin P., Duchoslav E., Chen K., Chelur A., Loyd B., Chernushevich I. (2021). Dissociation of Biomolecules by an Intense Low-Energy Electron Beam in a High Sensitivity Time-of-Flight Mass Spectrometer. J. Am. Soc. Mass Spectrom..

[68] Carracedo A., Cantley L.C., Pandolfi P.P. (2013). Cancer metabolism: Fatty acid oxidation in the limelight. Nat. Rev. Cancer.

[69] Manzo T., Prentice B.M., Anderson K.G., Raman A., Schalck A., Codreanu G.S., Lauson C.B.N., Tiberti S., Raimondi A., Jones M.A. (2020). Accumulation of long-chain fatty acids in the tumor microenvironment drives dysfunction in intrapancreatic cd8+ t cells. J. Exp. Med..

[70] Kim J.A. (2020). Peroxisome Metabolism in Cancer. Cells.

[71] Smith R.E., Lespi P., di Luca M., Bustos C., Marra F.A., de Alaniz M.J.T., Marra C.A. (2008). A reliable biomarker derived from plasmalogens to evaluate malignancy and metastatic capacity of human cancers. Lipids.

