# Supplementary material for: p73α1, an Isoform of the p73 Tumor Suppressor, Modulates Lipid Metabolism and Cancer Cell Growth via Stearoyl-CoA Desaturase-1
Source: Cells. 2022 Aug 13;11(16):2516. doi: 10.3390/cells11162516 (PMC9406568; doi:10.3390/cells11162516)
Supplement: Supplementary file 1 [file cells-11-02516-s001.zip › cells-1862339-supplementary.pdf]

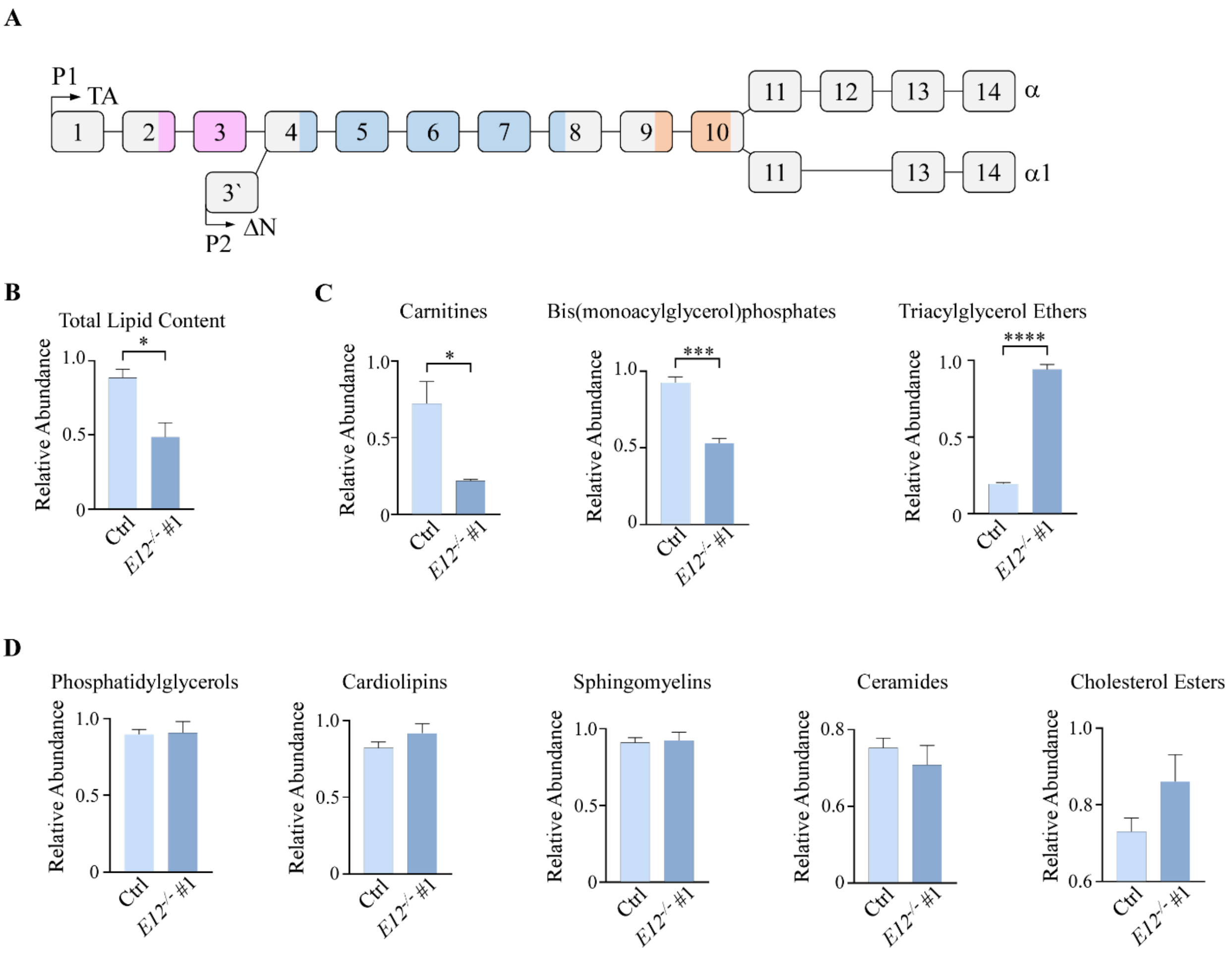

Figure S1. Loss of *E12* alters the lipidome in H1299 cells.

A. *TP73* diagram showing that loss of exon 12 leads to isoform switch from p73α to p73α1. Pink region indicates transactivation domain, blue region indicates DNA binding domain, and orange region indicates oligomerization domain. P1: promoter 1; P2: promoter 2.

B. Relative abundance of total lipid content in isogenic control and *E12*$^{-/-}$ H1299 cells. Statistical significance was determined using Student's t-test.

C. Relative abundance of carnitines, bis(monoacylglycerol)phosphates, and triacylglycerol ethers in isogenic control and *E12*$^{-/-}$ H1299 cells. Statistical significance was determined using Student's t-test.

D. Relative abundance of phosphatidylglycerols, cardiolipins, sphingomyelins, ceramides, and cholesterol esters in isogenic control and *E12*$^{-/-}$ H1299 cells. Statistical significance was determined using Student's t-test.

For B-D. Data are presented as mean ± SEM. n = 3 independent experiments. * $p < 0.05$, *** $p < 0.001$, **** $p < 0.0001$.

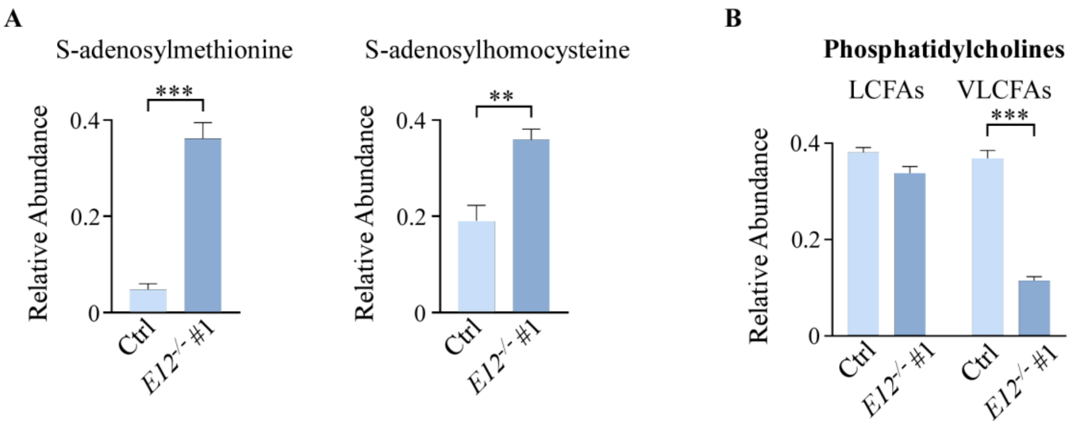

Figure S2. Metabolites involved in PC synthesis are altered upon loss of *E12*.

A. Relative abundance of S-adenosylmethionine and S-adenosylhomocysteine in isogenic control and $E12^{-/-}$ H1299 cell lines. Statistical significance was determined using Student's t-test.

B. Relative abundance of PCs containing (left) long-chain fatty acids (LCFAs) and (right) very long-chain fatty acids (VLCFAs) in isogenic control and $E12^{-/-}$ H1299 cell lines. Statistical significance was determined using Student's t-test.

For A-B. Data presented as mean ± SEM. n = 3 independent experiments. ** $p < 0.01$, *** $p < 0.001$.

A

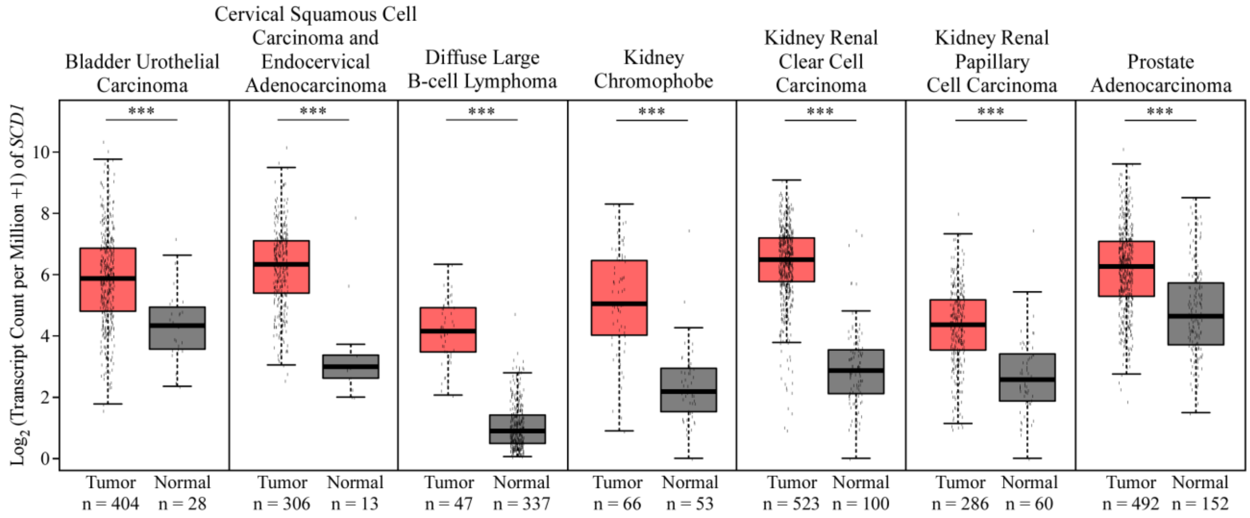

B

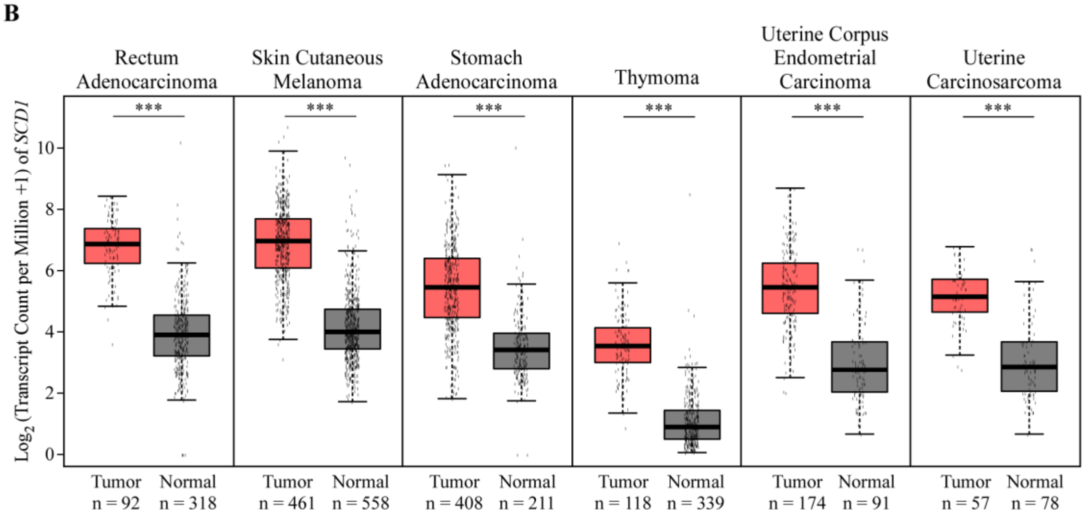

Figure S3. *SCD1* transcript counts are increased in certain cancer types.

A-B. *SCD1* transcript counts in the indicated tumors (data from TCGA) and the matched normal tissues (data from TCGA and GTEx) were analyzed via Gene Expression Profiling Interactive Analysis (GEPIA, http://gepia.cancer-pku.cn/index.html). *** $p < 0.001$.

Table S1. Primers used to generate expression vectors.

Name	Oligonucleotide	Sequence
p73-E12-gRNA-1-pSpCas9(BB)-2A-Puro	Sense	5`-CACCGCGTCACATCGCCAGGCCTT-3`
	Antisense	5`-AAACAAGGCCTGGCGATGTGACGC-3`
p73-E12-gRNA-2-pSpCas9(BB)-2A-Puro	Sense	5`-CACCGCTGCTGCTCATCTCGCCGT-3`
	Antisense	5`-AAACACGGCGAGATGAGCAGCAGC-3`

Table S2. Primers used for qPCR and ChIP.

TP73α1	Sense	5`-CACCGTCAAACGTGGTGCCCCCATC-3`
	Antisense	5`-AAACGATGGGGGCACCACGTTTGAC-3`
SCD1	Sense	5`-CACTTGGGAGCCCTGTATGG-3`
	Antisense	5`-TGAGCTCCTGCTGTTATGCC-3`
PCYT1A	Sense	5`-GAGTTGCAGCATGTGCCGA-3`
	Antisense	5`-GCATTGACCTTGGCTGAACA-3`
CEPT1	Sense	5`-GGCAGTGATTGGAGGACCAC-3`
	Antisense	5`-TGCCAACACCACCTGTGAAG-3`
SMPD4	Sense	5`-GACTCCCAGCCCCGGTGT-3`
	Antisense	5`-CCACTCGGAACACCATGAG-3`
SLC44A1	Sense	5`-GCAGAGCTCCAAACGAGAA-3`
	Antisense	5`-AGCCACAAATAAATCCCATCCC-3`
HPRT1	Sense	5`-CACCGCGTCACATCGCCAGGCCTT-3`
	Antisense	5'-AAACAAGGCCTGGCGATGTGACGC-3`
SCD1-ChIP	Sense	5`-TGCAGGGGTTTTTCGGAGTTT-3`
	Antisense	5`-TGAACGCCCTATTCCAGCCTTA-3`
CDKN1A-ChIP	Sense	5`-GGTCTGCTACTGTGTCCTCC-3`
	Antisense	5`-CATCTGAACAGAAATCCCAC-3`
GAPDH-ChIP	Sense	5`-AAAAGCGGGGAGAAAGTAGG-3`
	Antisense	5`-AAGAAGATGCGGCTGACTGT-3`
